# Leptin activates Akt in oesophageal cancer cells via multiple atorvastatin-sensitive small GTPases

**DOI:** 10.1007/s11010-021-04067-8

**Published:** 2021-02-13

**Authors:** Ian L. P. Beales, Olorunseun O. Ogunwobi

**Affiliations:** 1 Department of Gastroenterology, Norfolk and Norwich University Hospital, Norwich, NR4 7UZ UK; 2 Gastrioenterology Research Unit, Norwich Medical School, University of East Anglia, Norwich, NR4 7TJ UK

**Keywords:** Akt, Barrett’s oesophagus, Hydroxymethyl-CoA reductase inhibitor, Leptin, Monomeric GTP-binding proteins, Obesity

## Abstract

Obesity is a risk factor for Barrett’s oesophagus and oesophageal adenocarcinoma. Adipose tissue secretes the hormone leptin. Leptin is a growth factor for several cell types, including Barrett’s cells and oesophageal adenocarcinoma cells. Statins are associated with reduced rates of Barrett’s oesophagus and oesophageal cancer and exhibit anti-cancer effects in vitro. The mechanisms of these effects are not fully established. We have examined the effects of leptin and the lipid-soluble statin, atorvastatin, on signalling via monomeric GTP-binding proteins and Akt. Proliferation and apoptosis were assessed in OE33 cells. Akt activity was quantified by cell-based ELISA and in vitro kinase assay. Specific small-molecule inhibitors and a dominant-negative construct were used to reduce Akt activity. Small GTPases were inhibited using transfection of dominant-negative plasmids, prenylation inhibitors and pretreatment with atorvastatin. Leptin stimulated Akt activity and cell proliferation and inhibited camptothecin-induced apoptosis in an Akt-sensitive manner. Leptin induced phosphorylation of Bad and FOXO1 in an Akt-sensitive manner. Leptin activated Ras, Rac, RhoA and cdc42. Transfection of dominant-negative plasmids confirmed that leptin-induced Akt activation required Ras, RhoA cdc42 but not Rac. Atorvastatin inhibited leptin-induced activation of Ras, RhoA, cdc42 and Akt. Co-treatment with mevalonate prevented these effects of atorvastatin. The protein kinase Akt is essential to the growth-promoting and anti-apoptotic effects of leptin in oesophageal adenocarcinoma cells. Akt is activated via Ras-, Rho- and cdc42-dependant pathways. Atorvastatin reduces leptin-induced Akt activation by inhibiting prenylation of small GTPases. This may explain the reduced incidence of oesophageal adenocarcinoma in statin-users.

## Introduction

OAC is an important cause of mortality and morbidity. In the USA and Europe, incidence rates have increased by approximately 7-fold in the last 40 years [1]. Survival rates for established OAC are poor (5-year survival less than 20%) [2, 3]. In most cases, OAC is thought to develop from the precursor lesion, BO, which is a metaplastic phenotypic change in the oesophageal mucosa from squamous to glandular-type. Subsequently, cancer develops along a dysplasia-carcinoma sequence. The pathogenesis of BO and OAC is complex but the two most established modifiable risk factors for both are acid-reflux and obesity (particularly central obesity) [4]. The interaction of these is complex. Several lines of evidence of have suggested that actions and interactions between acid and peptides secreted by adipose tissue, at the level of the oesophageal epithelium, may promote the development of OAC in addition to the combination of effects of lifestyle and effects on the gastrooeosphageal junction [5–7].

We have demonstrated previously that the hormone leptin, produced by adipose tissue, has important effects in stimulating proliferation and inhibiting apoptosis in Barrett’s epithelial cells [8, 9]. Levels of leptin rise in proportion to adipose tissue mass [5]. Increased leptin levels are an independent risk factor for the development of both BO and progression along the dysplasia-carcinoma sequence [10, 11]. Functional leptin receptors are expressed on the mucosa of BO and cultured Barrett’s cell lines [5, 8, 9] Leptin is also secreted into the gastric lumen by gastric chief cells and theoretically Barrett’s mucosa is exposed to leptin from both the circulation and gastric refluxate [12, 13].

In experimental in vitro models, acid-reflux also stimulates proliferation and inhibits apoptosis in malignant and non-malignant Barrett’s cell lines [14, 15]; the combined effects of acid and leptin are synergistic [16]. Leptin has also been shown to increase the resistance of oesophageal cancer to chemotherapy in vivo and in vitro [17]. The mechanisms of these effects are incompletely defined.

The serine-threonine protein kinase Akt (also known as protein kinase B) is important in many systems in controlling proliferation and apoptosis. We have previously reported that Akt activation increases in Barrett’s mucosa along the non-dysplastic to low-grade dysplasia to high-grade dysplasia sequence. Akt is activated in oesophageal Barrett’s cells by acid-reflux [18] and by hormones such as gastrin and glycine-extended gastrin as well as leptin [7, 8, 19, 20]. In colon cancer cells, leptin-induced Akt activation is essential to the anti-apoptotic and proliferative actions [21]. Previously studies have outlined some of the pathways downstream of Akt that mediate the anti-apoptotic effects in Barrett’s epithelial cells but there are much less data on the mechanisms that are involved in the upstream pathways leading to activation of Akt in this system [18, 22]. Membrane-bound small GTP-binding proteins (GTPases) are important regulators of the signalling between cell-membrane receptors and intracellular signals, including protein kinases such as Akt. These GTP-binding proteins of the Ras, Rho, Rac and cdc42 subfamilies have been implicated in a variety of cellular processes including proliferation, cell-survival, invasion and inflammation [23]. Activation of the small GTP-binding proteins by both typical tyrosine kinase-linked growth factor receptors, including leptin [24, 25], and 7-transmembrane G-protein coupled receptors has been described [26–28]. Previously, Ras has been shown to mediate anti-apoptotic signalling in Barrett’s cells [29, 30], but the role of these GTP-binding proteins in OAC and Barrett’s mucosa have not been fully explored. We have also previously shown that statins (HMG-CoA reductase inhibitors) have anti-cancer effects in OAC cells in vitro [29] and are associated with a reduced incidence of OAC in epidemiological studies [31], and the mechanisms of these effects have not been fully described but may involve small GTPases.

Therefore, in this study, we have examined the hypothesis that GTP-binding proteins are involved in the regulation of Akt activation by leptin and that atorvastatin, a potent lipid-soluble statin, interferes with pro-oncogenic leptin signalling at the level of membrane-bound GTPases.

## Methods

### Cell culture, proliferation and apoptosis

OE33 OAC cells were cultured as described previously [8, 32]. For proliferation experiments, cells were serum-starved for 24 h before stimulation with recombinant human leptin (1nM) (Bachem, UK), and then proliferation was assessed using a BrdU incorporation assay as previously described [20]. Apoptosis was induced by exposing serum-free leptin-treated cells with camptothecin (Merck, UK), and apoptosis quantified with the ApoPercentage assay as described previously [19]. We have previously confirmed that these assays provide comparable data to that obtained by cell counting and the MTT assay [8, 9] and caspase-3 activity and quantification of intracellular nucleosomes [16, 19]. For inhibitor studies, the Akt inhibitors, API-2 and GSK690693 (both 1 μm) [33, 34] (Tocris, Abingdon, UK), were added 60 min prior to leptin. Cells were treated with adenoviral vector containing DN-Akt (Vector Biolabs, Pennsylvania, USA) 24 h prior to stimulation with leptin as described [26].

#### Akt activation and downstream activity

Serum-starved OE33 cells were stimulated with leptin, subsequently phosphorylated and non-phosphorylated Akt, Bad and FOXO1 (Forkhead box O1) were quantified in formalin-fixed cells using commercially-available cell-based ELISAs (Active Motif, Waterloo, Belgium) as described previously [18]. Akt activity was additionally measured using an in vitro kinase activity as described previously [20].

#### Assessment of the on involvement of small GTPases

OE33 cells were treated 24 h before leptin stimulation with expression plasmids containing DN-Ras, DN-cdc42, DN-RhoA and DN-Rac1 (cDNA Resource Centre, Bloomsburg University, Pennsylvania, USA), using previously described methodology [19]. Where appropriate, the prenylation inhibitors FTI276 and GGTI298 (both 10 μM) (both Tocris, Abingdon, Oxford, UK) were added 24 h prior to leptin stimulation [29]. In selected experiments, atorvastatin (0.1 μM) (Tocris) and mevalonate (100 μm) (Sigma) were initially added 72 h prior to stimulation with leptin, with a medium change with fresh reagents added after 48 h.

#### Assessment of GTPase activity

Activity of Ras, cdc42, RhoA and Rac1,2,3 in leptin-stimulated OE33 cell lysates was quantified using G-LISA colormetric assays, according to the manufacturer’s instructions (Cytoskeleton, Inc, Denver, CO, USA).

## Results

### Inhibition of Akt reduces leptin-stimulated proliferation and evasion of apoptosis

**Fig. 1:**
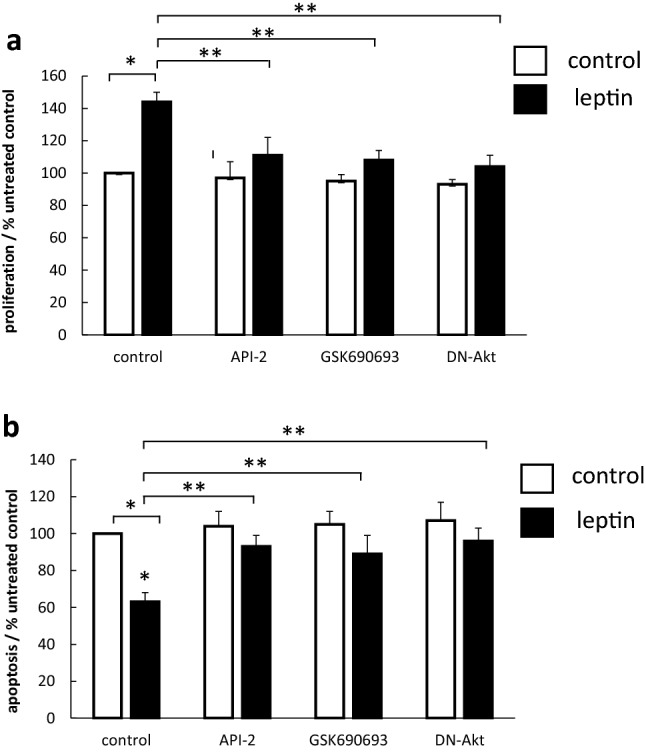
Effect of Akt inhibitors on the pro-proliferative and anti-apoptotic effects of leptin in OE33 cells. (**a**) Effects on proliferation. Serum-starved OE33 cells were pretreated where appropriate before stimulation with leptin (1 nM) with the Akt inhibitors API-2 or GSK690693 (both 1 μM), 60 min before leptin or a dominant-negative adenoviral construct (DN-Akt) 24 h pre-leptin. Cell proliferation after 24 h was measured using a specific BrdU incorporation ELISA. Results are expressed as mean ± SEM, N = 4, *P < 0.01 vs control unstimulated, **P < 0.01 vs leptin-stimulated. (**b**) Effects on apoptosis. OE33 cells were cultured for 42 h in serum-free medium or supplemented with 1 nM leptin. Camptothecin 10 μM was then added and apoptosis after a further 6 h quantified with the Biocolor APOPercentage™ assay. Results expressed as mean ± SEM, N = 4, *P < 0.01 vs camptothecin-treated control, **P < 0.01 vs leptin-treated

Leptin stimulated proliferation of OE33 cells, and this was significantly blocked (>80%) by 2 separate Akt-specific inhibitors and transfection with a DN-Akt construct (Fig. 1a). Leptin also demonstrated a significant anti-apoptotic effect. Leptin reduced camptothecin-induced apoptosis by 40%. Pretreatment with both Akt inhibitors and the DN-Akt plasmid abolished this anti-apoptotic effect (Fig. 1b).

### Leptin-stimulated Akt is enzymatically active and leads to downstream signalling

**Fig. 2:**
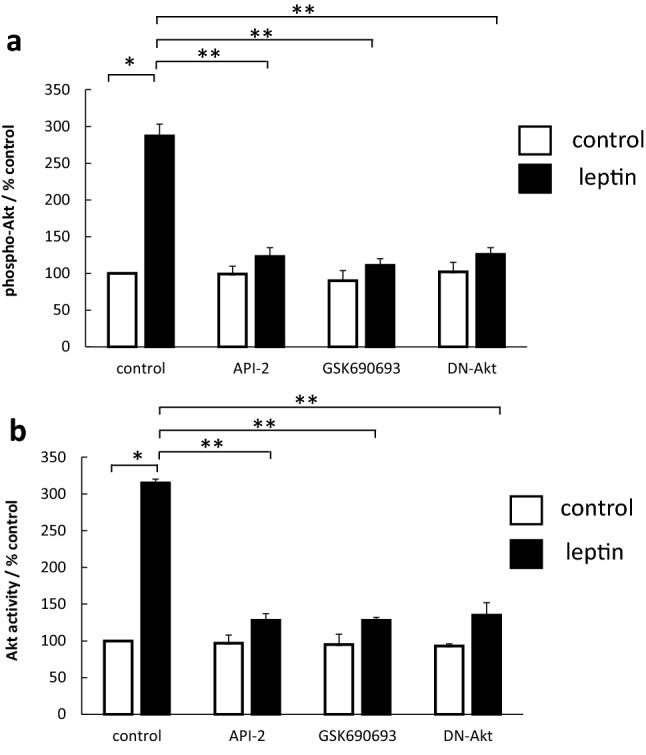
Effect of leptin on activation of Akt. Serum-starved OE33 cells were stimulated with leptin 1 nM. Where indicated inhibitors (API-2 1 μm, GSK690693 1 μM) were added 60 min prior to leptin or a dominant-negative Akt adenoviral construct (DN-Akt) added 24 h prior to leptin. (**a**) Effect on Akt phosphorylation. After 5 min, cells were formalin-fixed and subsequently phosphorylated Akt and total Akt levels quantified in fixed cells using a specific cell-based ELISA. Results for each well were normalised for cell content using subsequent crystal violet staining. Results are expressed as the ratio of phosphorylated/total kinase relative to control-treated cells. (**b**) Effect on Akt activity. After 5 min, cells were lysed and Akt kinase activity quantified in Akt immunoprecipitates using a specific kinase (K-LISA) kit. Results are expressed as phosphorylating activity compared to untreated controls. Results are expressed as mean ± SEM, N = 5, *P < 0.01 vs control untreated, **P < 0.01 vs leptin-treated

The activation of Akt by leptin was confirmed using 2 different assays. Leptin-stimulated Akt activation was assessed by assay measuring Akt phosphorylation (increased by 187%) as well as by a specific kinase assay (increased by 215%). Pretreatment with Akt inhibitors or the DN-Akt effectively blocked Akt activation, with similar effects in both assays (Fig. 2). Total Akt levels were unaltered by leptin or small-molecule inhibitors (data not shown), consistent with previously published data [7, 8, 16].

**Fig. 3:**
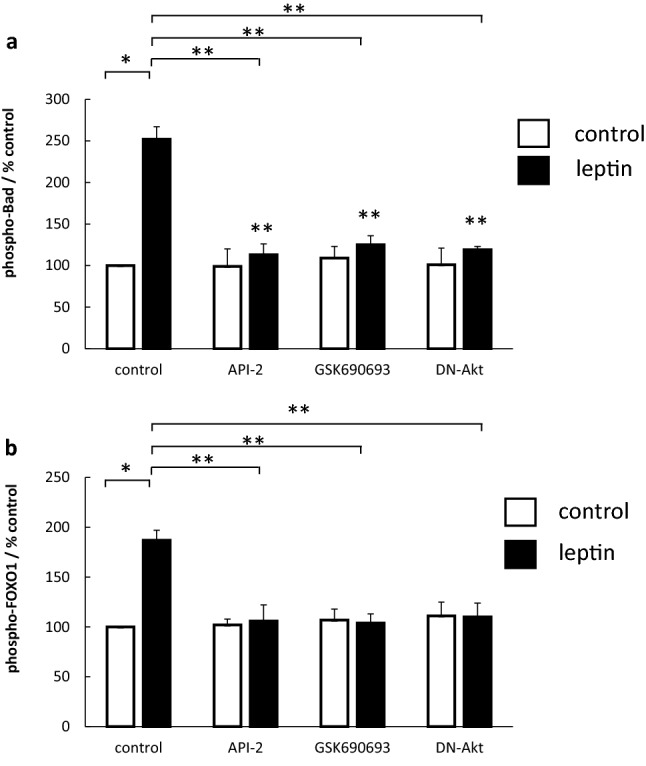
Effect Akt inhibitors on leptin-stimulated phosphorylation of Bad and FOXO1. Serum-starved OE33 cells were stimulated with leptin 1 nM. Where indicated inhibitors (API-2 1 μm, GSK690693 1 μM) were added 60 min prior to leptin or a DN-Akt adenoviral construct added 24 h prior to leptin. After 5 min, cells were formalin-fixed and phosphorylated and non-phosphorylated proteins assessed by specific cell-based ELISAs. (**a**) Effect on Bad-phosphorylation, (**b**) Effect on FOXO1-phosphorylation. Results for each well were normalised for cell content using subsequent crystal violet staining. Results are expressed as the ratio of phosphorylated/total protein relative to control-treated cells. Results are expressed as mean ± SEM, N = 3, *P < 0.01 vs control untreated, **P < 0.05 vs leptin-treated

To confirm that leptin-stimulated Akt was functionally active, we further assessed downstream markers of Akt activity as read-outs. Leptin stimulation led to an increase in the phosphorylation of both Bad (increased by 150%) (Fig. 3a) and FOXO1 (increased by 87%) (Fig. 3a, b) and in both cases this was significantly reduced by the Akt inhibitors and the DN-Akt construct.

### Akt activation by leptin requires small GTP-binding protein activity

**Fig. 4:**
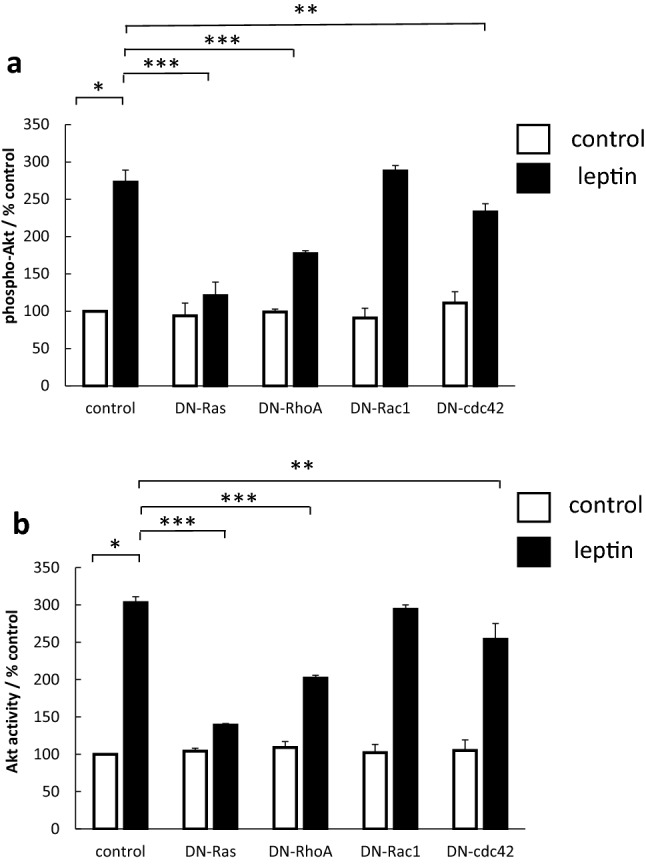
Effect of inhibition of small GTP-binding proteins on leptin-stimulated Akt activity. OE33 cells were transfected with expression plasmids containing DN-Ras, DN-RhoA, DN-Rac1 or DN-cdc42. After 24 h serum-starvation, cells were stimulated with 1 nM leptin. (**a**) Effect on Akt phosphorylation. After 5 min, cells were formalin-fixed and subsequently phosphorylated Akt and total Akt levels quantified in fixed cells using a specific cell-based ELISA. Results for each well were normalised for cell content using subsequent crystal violet staining. Results are expressed as the ratio of phosphorylated/total kinase relative to control-treated cells. (**b**) Effect on Akt activity. After 5 min, cells were lysed and Akt kinase activity quantified in Akt immunoprecipitates using a specific kinase (K-LISA) kit. Results are expressed as phosphorylating activity compared to untreated controls. Results are expressed as mean ± SEM, N = 5, *P < 0.01 vs control untreated, **P < 0.05 vs leptin-treated, ***P < 0.01 vs leptin-treated

The activation of protein kinases is controlled in many cases by small GTP-binding protein. We examined the role of the small GTP-binding proteins Ras, Rac, Rho and cdc42 in leptin-stimulated Akt activation. Transfection of OE33 cells with DN-mutants of the different GTPases had different actions on Akt activation. DN-Ras inhibited Akt activation by >80%, DN-RhoA by 50–57% and DN-cdc42 by about 25%. DN-Rac1 had no effect on Akt activation. A similar pattern of results was seen whether Akt was assessed with either a cell-based ELISA for phosphorylated protein (Fig. 4a) or in a kinase assay (Fig. 4b).

**Fig. 5:**
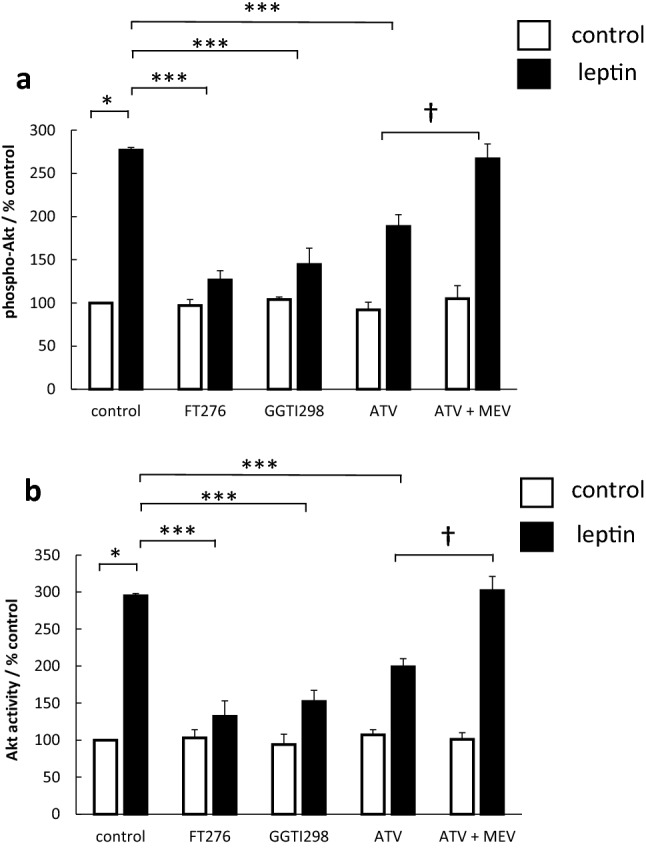
Effect of prenylation inhibitors on leptin-stimulated Akt activity. Serum-starved OE33 cells were stimulated with 1 nM leptin. Either 24 h prior to leptin stimulation cells were treated with the farnesylation inhibitor FT276 (10 μM) or the geranylgeranylation inhibitor GGT298 (10 μM) or 72 h prior to stimulation treated with atorvastatin (0.1 μM) (ATV) with or without mevalonate (100 μM) (MEV). (**a**) Effect on Akt phosphorylation. After 5 min, leptin stimulation cells were formalin-fixed and subsequently phosphorylated Akt and total Akt levels quantified in fixed cells using a specific cell-based ELISA. Results for each well were normalised for cell content using subsequent crystal violet staining. Results are expressed as the ratio of phosphorylated/total kinase relative to control-treated cells. (**b**) Effect on Akt activity. After 5 min, leptin stimulation cells were lysed and Akt kinase activity quantified in Akt immunoprecipitates using a specific kinase (K-LISA) kit. Results are expressed as phosphorylating activity compared to untreated controls. Results are expressed as mean ± SEM, N = 5, *P < 0.01 vs control untreated, ***P < 0.01 vs leptin-treated, ^†^*P* < 0.05 vs atorvastatin-treated

Small GTP-binding protein is active when localised to the cell membrane; this localisation requires post-translational modification by prenylation with the addition of a farnesyl group (to Ras) and geranylgeranyl groups to Rac, Rho and cdc42). Therefore, we performed further studies using specific inhibitors of farnesylation and geranylgeranylation. The farnesyl transferase inhibitor FTI276 reduced leptin-stimulated Akt activation by 85% and the geranylgeranyl transferase inhibitor GGTI298 reduced Akt activation by approximately 75%. Statins reduce the ability to prenylate GTP-binding protein by reducing the availability of farnesyl and geranylgeranyl intermediates subsequent to the inhibition of the mevalonate synthetic pathway [35]. Pretreatment with the lipid-soluble statin, atorvastatin, also inhibited Akt activation by 50%. Co-treatment with mevalonate prevented this inhibitory effect (Fig. 5).

### Leptin stimulates small GTP-binding protein activity

**Fig. 6:**
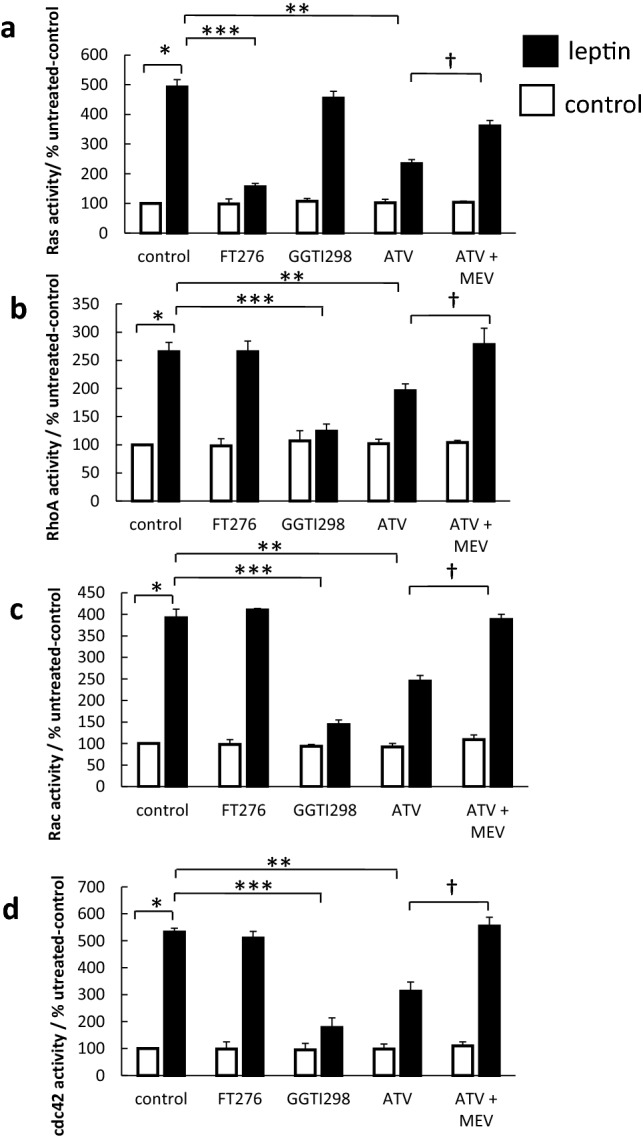
Effect of prenylation inhibitors on small GTPase activity. Serum-starved OE33 cells were stimulated with 1 nM leptin. Either 24 h prior to leptin stimulation cells were treated with the farnesylation inhibitor FT276 (10 mM) or the geranylgeranylation inhibitor GGT298 (10 mM) or 72 h prior to stimulation treated with atorvastatin (0.1 mM) (ATV) with or without mevalonate (100 mM) (MEV). Three minutes after, leptin stimulation cells were harvested and the activity of small GTPase was assessed. (**a**) Ras activity. (**b**) Rho activity. (**c**) Rac activity and (**d**) cdc42 activity. Results are expressed as mean ± SEM, N = 4, *P < 0.01 vs control untreated, **P < 0.05 vs leptin-treated, *** *P* < 0.01 vs leptin-treated, ^†^*P* < 0.05 vs atorvastatin-treated

To confirm the involvement of small GTPase in leptin signalling, the activity of all 4 classes of GTPase was assessed using specific assays. Leptin stimulation potently increased the activity of Ras, Rac, Rho and cdc42 (Fig. 6). The farnesyltransfer inhibitor inhibited the activity of Ras, whilst the geranylgeranylation inhibitor reduced the activation of Rac, Rho and cdc42. Pretreatment with atorvastatin reduced leptin-stimulated activation of all classes of GTP-binding protein (by 54–76%) and co-treatment with mevalonate significantly ameliorated this effect in all cases.

## Conclusions

In this study, we have demonstrated for the first time the essential requirement for the small GTPases Ras, Rho and cdc42 in leptin-induced Akt activation in oesophageal cancer cells. We have also shown that the lipid-soluble statin, atorvastatin, inhibits leptin-induced pro-oncogenic signalling by reducing signalling via these membrane-linked GTPases.

We have confirmed that leptin exerts important pro-proliferative and anti-apoptotic effects on OAC cells. We have demonstrated the essential role of the protein kinase Akt in this pathway. We confirmed increased leptin-stimulated Akt activity using two separate methods. Similar effects were seen using 3 different methods of inhibiting Akt. Although the two different small-molecule Akt inhibitors used have been reported to be Akt selective, most kinase inhibitors are not completely specific; hence, we performed confirmatory experiments using transfection with a dominant-negative adenoviral construct. All these results were consistent and clearly implicate Akt as a downstream mediator in the effects of leptin in oesophageal cancer cells.

The specific downstream effectors of the Akt activity in this model remain to be clarified. Important actions of Akt include phosphorylation of Forkhead (FOXO) transcription factors, which leads to subsequent sequestration in the cytosol and inhibition of transcriptional activity and phosphorylation and reduced activity of the proapoptotic protein Bad. These have been implicated in the anti-apoptotic effect mechanisms of other signalling systems, such as that induced by gastrin [36]. We used these two markers primarily as read-outs to confirm the functional activity of Akt in OE33 cells. Our results confirm the functional enzymatic activity of Akt in the system, although, at present, we cannot definitely implicate either Bad or FOXO1 as downstream mediators of Akt. FOXO1 is known to be an essential downstream mediator of the central anorectic effects of activation of the leptin receptor [37]. Other downstream targets of Akt, such as the NF-κB pathway and stabilisation of COX-2 mRNA, are likely to be important in the anti-apoptotic effects of Akt in Barrett’s oesophageal cells [19, 20]. Recent studies have also further highlighted the potential central role of Akt in Barrett’s oesophageal cancer progression: signalling via the insulin/insulin-like growth factor-1 axis and HER2 receptor also activates Akt along the Barrett’s dysplasia-cancer sequence [38]. This latter study did not examine the upstream activators of Akt or the downstream effectors.

Our results show that activation of the leptin receptor activates small GTP-binding proteins of all 4 subfamilies studied (Ras, Rho, Rac and cdc42). The data from the dominant-negative transfection studies clearly demonstrated that although leptin-induced Akt activation was independent of Rac activation, there was a requirement for all three other GTP-binding protein families. Inhibition of Ras had by far the greatest inhibitory effect on leptin-induced Akt activity, and there were smaller effects from inhibiting RhoA and cdc42. These data show that leptin-induced Akt activation involves Ras, Rho and cdc42. There did not seem to be an absolute requirement for any specific GTP-binding protein, although Ras seemed to have the dominant role in signalling. Previously, leptin has been shown to activate all of Ras, Rho, Rac and cdc42 in a variety cell models, although the exact significance of these changes and the downstream effectors have not been fully described. In colon cancer cells, leptin activates Rho, Rac and cdc42 which are involved in enhancing migration and invasion [24, 39]. There are a wealth of data examining downstream signalling from the leptin receptor, this is concentrated on protein kinases and transcription factors and there are relatively few studies examining the specific role of the small GTPase families [40].

We further explored different pharmacological methods to manipulate GTP-binding protein signalling and the effects on Akt activation. The results with the prenylation inhibitors were consistent with the dominant-negative plasmids. Ras is predominantly farnesylated, whilst Rho, Rac and cdc42 are geranylgeranylated [35]. The addition of the lipid tails allows localisation of the GTP-binding protein to the inner side of the cell membrane, where they are functionally involved in signalling from many receptors. The farnesyl transferase inhibitor reduced Akt activation more than the geranylgeranyl transferase inhibitor, in keeping both with the dominant role of Ras in leptin-induced Akt activation, and the subsidiary but important roles of Rho and cdc42.

Previous studies, both from our own group and others, have shown that statins have anti-proliferative and proapoptotic actions in oesophageal adenocarcinoma cells [29, 41, 42]. Clinical epidemiological studies have shown associations between statin use and reduced incidences of both BO and OAC [43–46] and improved overall and cancer-specific survival after surgery for OAC [47], suggesting that these effects of statins do have clinical correlations. However, the exact mechanism(s) underlying these apparent protective associations are not fully defined [5], but the clinical and experimental data do support further the further exploration of statins as chemopreventative agents in BO.

Statins inhibit the essential early step in the pathway for the formation of mevalonate. Mevalonate is the precursor to cholesterol but also the precursor to farnesyl pyrophosphate and geranylgeranyl pyrophosphate, which are the required substrates for the prenylation and membrane localisation of small GTP-binding proteins. We pretreated OE33 cells with the lipid-soluble statin atorvastatin at approximately the same concentration as can be found with high-dose clinical use (40–80 mg daily) [48, 49]. Atorvastatin significantly reduced by Akt activation and GTP-binding protein activity. These effects of atorvastatin were abolished by adding back mevalonate. Mevalonate is downstream of HMG-CoA reductase in the biosynthetic pathways and is the precursor farnesyl and geranylgeranyl pyrophosphate, suggesting that the effects of atorvastatin were mediated by the reduction in the availability of intermediates for prenylation and resultant reduction in GTP-binding protein signalling.

Although potentially useful anti-cancer effects of statins in vitro against oesophageal cancer cells and non-malignant Barrett’s oesophageal cells have been documented, consistent with the clinical observations, some of these studies have been criticised for using what were clearly supraphysiological statin concentrations [50]. In this current study, we specifically used atorvastatin concentrations that can be achieved with standard dosing regimens [48, 49]. The inhibitory effects of atorvastatin demonstrated in this study were clearly less than those obtained with the more specific experimental tools to inhibit Akt and small GTP-binding proteins (such as the dominant-negative constructs and prenylation inhibitors). This presumably reflects the less than total inhibition of the mevalonate pathway possible with pharmacologically feasible statin levels: even then this atorvastatin concentration is well over the IC_50_ for HMG-CoA-reductase (15.2 nM [51]. Higher degrees of apoptosis and inhibition of proliferation have been achieved using considerably higher concentrations of statins (up to 50 μm) [41, 42]. The  specificity of the pharmacological effects and clinical relevance of these effects are open to question.

Recently, alternative methods of inhibition of small GTPase signalling by statins have been described: atorvastatin and pitavastatin inhibited Rac signalling by enhancing degradation of Rac in a prenylation-independent manner [52]. In our current study, the effects of atorvastatin were reversed by mevalonate favouring a prenylation-dependant pathway.

The effects of statins in potentially preventing the development or progression of OAC in vivo are undoubtedly complex, but our current data suggest that direct effects at the level of the oesophageal epithelial cells may be involved. Although the experimental cell-line studies implicating leptin signalling, GTPases and the ameliorating effect of statins [29, 53] are all consistent with the epidemiological and clinical studies [43, 54–56], these particular pathways have not been examined as yet in the animal models of Barrett’s carcinogenesis [57].

Similarly, the mechanisms linking obesity and the development of OAC are complex. Direct effects of adipose tissue mediators such as leptin are but one of the likely factors including lifestyle, environment and disruption of the anti-reflux barrier [5, 6]. However, our studies further confirm that leptin can have important pro-neoplastic effects directly on the oesophageal glandular epithelium. The protein kinase Akt has an essential role in these leptin-mediated effects and the small GTP-binding proteins Ras, Rho and cdc42 are involved in regulation Akt activity. Further studies examining the pharmacological modulation of Akt in oesophageal neoplasia and pre-neoplasia are warranted. Statins, in this case atorvastatin, by affecting GTP-binding protein prenylation and activation can reduce Akt activation and the potential role of statins to prevent OAC should be explored further.

## Data Availability

Data archiving is not mandated but data will be made available on reasonable request.

## Abbreviations

BO: Barrett’s oesophagus
DN-Akt: Dominant-negative Akt
ELISA: Enzyme-linked immunoabsorbant assay
GTP: Guanosine triphosphate
HMG-CoA: 3-hydroxy-3-methyl-glutaryl-coenzyme
MTT: 3-(4,5-dimethylthiazol-2-yl)-2,5-diphenyltetrazolium bromide
NF-kB: Nuclear factor kappa B
OAC: Oesophageal adenocarcinoma

## References

[1] Runge TM, Abrams JA, Shaheen NJ (2015). Epidemiology of barrett’s esophagus and esophageal adenocarcinoma. Gastroenterol Clin N Am.

[2] Njei B, McCarty TR, Birk JW (2016). Trends in esophageal cancer survival in United States adults from 1973 to 2009: a SEER database analysis. J Gastroenterol Hepatol.

[3] Dong J, Gu X, El-Serag HB, Thrift AP (2018). Underuse of surgery accounts for racial disparities in esophageal cancer survival times: a matched cohort study. Clin Gastroenterol Hepatol.

[4] Spechler SJ, Souza RF (2014). Barrett’s esophagus. N Engl J Med.

[5] Long E, Beales IL (2014). The role of obesity in oesophageal cancer development. Ther Adv Gastroenterol.

[6] Alexandre L, Long E, Beales IL (2014). Pathophysiological mechanisms linking obesity and esophageal adenocarcinoma. World J Gastrointest Pathophysiol.

[7] Ogunwobi OO, Beales IL (2008). Globular adiponectin, acting via adiponectin receptor-1, inhibits leptin-stimulated oesophageal adenocarcinoma cell proliferation. Mol Cell Endocrinol.

[8] Ogunwobi O, Mutungi G, Beales IL (2006). Leptin stimulates proliferation and inhibits apoptosis in Barrett’s esophageal adenocarcinoma cells by cyclooxygenase-2-dependent, prostaglandin-E2-mediated transactivation of the epidermal growth factor receptor and c-Jun NH2-terminal kinase activation. Endocrinology.

[9] Ogunwobi OO, Beales IL (2008). Leptin stimulates the proliferation of human oesophageal adenocarcinoma cells via HB-EGF and Tgfalpha mediated transactivation of the epidermal growth factor receptor. Br J Biomed Sci.

[10] Duggan C, Onstad L, Hardikar S, Blount PL, Reid BJ, Vaughan TL (2013). Association between markers of obesity and progression from Barrett’s esophagus to esophageal adenocarcinoma. Clin Gastroenterol Hepatol.

[11] Chandar AK, Devanna S, Lu C, Singh S, Greer K, Chak A, Iyer PG (2015). Association of serum levels of adipokines and insulin with risk of Barrett’s esophagus: a systematic review and meta-analysis. Clin Gastroenterol Hepatol.

[12] Mix H, Widjaja A, Jandl O, Cornberg M, Kaul A, Goke M, Beil W, Kuske M, Brabant G, Manns MP, Wagner S (2000). Expression of leptin and leptin receptor isoforms in the human stomach. Gut.

[13] Cammisotto PG, Renaud C, Gingras D, Delvin E, Levy E, Bendayan M (2005). Endocrine and exocrine secretion of leptin by the gastric mucosa. J Histochem Cytochem.

[14] Huo X, Zhang X, Yu C, Cheng E, Zhang Q, Dunbar KB, Pham TH, Lynch JP, Wang DH, Bresalier RS, Spechler SJ, Souza RF (2018). Aspirin prevents NF-kappaB activation and CDX2 expression stimulated by acid and bile salts in oesophageal squamous cells of patients with Barrett’s oesophagus. Gut.

[15] Sarosi GA, Jaiswal K, Herndon E, Lopez-Guzman C, Spechler SJ, Souza RF (2005). Acid increases MAPK-mediated proliferation in Barrett’s esophageal adenocarcinoma cells via intracellular acidification through a Cl-/HCO3-exchanger. Am J Physiol Gastrointest Liver Physiol.

[16] Beales IL, Ogunwobi OO (2007). Leptin synergistically enhances the anti-apoptotic and growth-promoting effects of acid in OE33 oesophageal adenocarcinoma cells in culture. Mol Cell Endocrinol.

[17] Bain GH, Collie-Duguid E, Murray GI, Gilbert FJ, Denison A, McKiddie F, Ahearn T, Fleming I, Leeds J, Phull P, Park K, Nanthakumaran S, Matula KM, Grabsch HI, Tan P, Welch A, Schweiger L, Dahle-Smith A, Urquhart G, Finegan M, Petty RD (2014). Tumour expression of leptin is associated with chemotherapy resistance and therapy-independent prognosis in gastro-oesophageal adenocarcinomas. Br J Cancer.

[18] Beales IL, Ogunwobi O, Cameron E, El-Amin K, Mutungi G, Wilkinson M (2007). Activation of Akt is increased in the dysplasia-carcinoma sequence in Barrett’s oesophagus and contributes to increased proliferation and inhibition of apoptosis: a histopathological and functional study. BMC Cancer.

[19] Beales IL, Ogunwobi OO (2009). Glycine-extended gastrin inhibits apoptosis in Barrett’s oesophageal and oesophageal adenocarcinoma cells through JAK2/STAT3 activation. J Mol Endocrinol.

[20] Ogunwobi OO, Beales IL (2008). Glycine-extended gastrin stimulates proliferation via JAK2- and Akt-dependent NF-kappaB activation in Barrett’s oesophageal adenocarcinoma cells. Mol Cell Endocrinol.

[21] Ogunwobi OO, Beales IL (2007). The anti-apoptotic and growth stimulatory actions of leptin in human colon cancer cells involves activation of JNK mitogen activated protein kinase, JAK2 and PI3 kinase/Akt. Int J Color Dis.

[22] Beales IL, Ogunwobi O (2006). Glycine-extended gastrin inhibits apoptosis in colon cancer cells via separate activation of Akt and JNK pathways. Mol Cell Endocrinol.

[23] Cardama GA, Gonzalez N, Maggio J, Menna PL, Gomez DE (2017). Rho GTPases as therapeutic targets in cancer (Review). Int J Oncol.

[24] Attoub S, Noe V, Pirola L, Bruyneel E, Chastre E, Mareel M, Wymann MP, Gespach C (2000). Leptin promotes invasiveness of kidney and colonic epithelial cells via phosphoinositide 3-kinase-, rho-, and rac-dependent signaling pathways. FASEB J.

[25] Valle A, Sastre-Serra J, Pol C, Miro AM, Oliver J, Roca P (2011). Proteomic analysis of MCF-7 breast cancer cell line exposed to leptin. Anal Cell Pathol (Amsterdam).

[26] Stepan V, Ramamoorthy S, Pausawasdi N, Logsdon CD, Askari FK, Todisco A (2004). Role of small GTP binding proteins in the growth-promoting and antiapoptotic actions of gastrin. Am J Physiol Gastrointest Liver Physiol.

[27] Nuche-Berenguer B, Jensen RT (2015). Gastrointestinal hormones/neurotransmitters and growth factors can activate P21 activated kinase 2 in pancreatic acinar cells by novel mechanisms. Biochim Biophys Acta.

[28] Notcovich C, Diez F, Tubio MR, Baldi A, Kazanietz MG, Davio C, Shayo C (2010). Histamine acting on H1 receptor promotes inhibition of proliferation via PLC, RAC, and JNK-dependent pathways. Exp Cell Res.

[29] Ogunwobi OO, Beales IL (2008). Statins inhibit proliferation and induce apoptosis in Barrett’s esophageal adenocarcinoma cells. Am J Gastroenterol.

[30] Yu C, Huo X, Agoston AT, Zhang X, Theiss AL, Cheng E, Zhang Q, Zaika A, Pham TH, Wang DH, Lobie PE, Odze RD, Spechler SJ, Souza RF (2015). Mitochondrial STAT3 contributes to transformation of Barrett’s epithelial cells that express oncogenic Ras in a p53-independent fashion. Am J Physiol Gastrointest Liver Physiol.

[31] Beales IL, Vardi I, Dearman L, Broughton T (2013). Statin use is associated with a reduction in the incidence of esophageal adenocarcinoma: a case control study. Dis Esophagus.

[32] Beales ILP, Garcia-Morales C, Ogunwobi OO, Mutungi G (2014). Adiponectin inhibits leptin-induced oncogenic signalling in oesophageal cancer cells by activation of PTP1B. Mol Cell Endocrinol.

[33] Yang L, Dan HC, Sun M, Liu Q, Sun XM, Feldman RI, Hamilton AD, Polokoff M, Nicosia SV, Herlyn M, Sebti SM, Cheng JQ (2004). Akt/protein kinase B signaling inhibitor-2, a selective small molecule inhibitor of Akt signaling with antitumor activity in cancer cells overexpressing Akt. Cancer Res.

[34] Levy DS, Kahana JA, Kumar R (2009). AKT inhibitor, GSK690693, induces growth inhibition and apoptosis in acute lymphoblastic leukemia cell lines. Blood.

[35] Yeganeh B, Wiechec E, Ande SR, Sharma P, Moghadam AR, Post M, Freed DH, Hashemi M, Shojaei S, Zeki AA, Ghavami S (2014). Targeting the mevalonate cascade as a new therapeutic approach in heart disease, cancer and pulmonary disease. Pharmacol Ther.

[36] Ramamoorthy S, Stepan V, Todisco A (2004). Intracellular mechanisms mediating the anti-apoptotic action of gastrin. Biochem Biophys Res Commun.

[37] Yang G, Lim CY, Li C, Xiao X, Radda GK, Li C, Cao X, Han W (2009). FoxO1 inhibits leptin regulation of pro-opiomelanocortin promoter activity by blocking STAT3 interaction with specificity protein 1. J Biol Chem.

[38] Arcidiacono D, Antonello A, Fassan M, Nucci D, Morbin T, Agostini M, Nitti D, Rugge M, Alberti A, Battaglia G, Realdon S (2017). Insulin promotes HER2 signaling activation during Barrett’s Esophagus carcinogenesis. Dig Liver Dis.

[39] Jaffe T, Schwartz B (2008). Leptin promotes motility and invasiveness in human colon cancer cells by activating multiple signal-transduction pathways. Int J Cancer.

[40] Wauman J, Zabeau L, Tavernier J (2017). The leptin receptor complex: heavier than expected?. Front Endocrinol (Lausanne).

[41] Konturek PC, Burnat G, Hahn EG (2007). Inhibition of Barret’s adenocarcinoma cell growth by simvastatin: involvement of COX-2 and apoptosis-related proteins. J Physiol Pharmacol.

[42] Sadaria MR, Reppert AE, Yu JA, Meng X, Fullerton DA, Reece TB, Weyant MJ (2011). Statin therapy attenuates growth and malignant potential of human esophageal adenocarcinoma cells. J Thorac Cardiovasc Surg.

[43] Thomas T, Loke Y, Beales ILP (2017). Systematic review and meta-analysis: use of statins is associated with a reduced incidence of oesophageal adenocarcinoma. J Gastrointest Cancer.

[44] Beales IL, Dearman L, Vardi I, Loke Y (2016). Reduced risk of Barrett’s esophagus in statin users: case-control study and meta-analysis. Dig Dis Sci.

[45] Kastelein F, Spaander MC, Biermann K, Steyerberg EW, Kuipers EJ, Bruno MJ (2011). Nonsteroidal anti-inflammatory drugs and statins have chemopreventative effects in patients with Barrett’s esophagus. Gastroenterology.

[46] Nguyen DM, Richardson P, El-Serag HB (2010). Medications (NSAIDs, statins, proton pump inhibitors) and the risk of esophageal adenocarcinoma in patients with Barrett’s esophagus. Gastroenterology.

[47] Alexandre L, Clark AB, Bhutta HY, Chan SS, Lewis MP, Hart AR (2016). Association between statin use after diagnosis of esophageal cancer and survival: a population-based cohort study. Gastroenterology.

[48] Corsini A, Bellosta S, Baetta R, Fumagalli R, Paoletti R, Bernini F (1999). New insights into the pharmacodynamic and pharmacokinetic properties of statins. Pharmacol Ther.

[49] Partani P, Verma SM, Gurule S, Khuroo A, Monif T (2014). Simultaneous quantitation of atorvastatin and its two active metabolites in human plasma by liquid chromatography/(-) electrospray tandem mass spectrometry. J Pharm Anal.

[50] Beales IL (2012). Letter: potential chemopreventive effects of statins in oesophageal adenocarcinoma. Aliment Pharmacol Ther.

[51] Poli A (2007). Atorvastatin: pharmacological characteristics and lipid-lowering effects. Drugs.

[52] Tanaka S, Fukumoto Y, Nochioka K, Minami T, Kudo S, Shiba N, Takai Y, Williams CL, Liao JK, Shimokawa H (2013). Statins exert the pleiotropic effects through small GTP-binding protein dissociation stimulator upregulation with a resultant Rac1 degradation. Arterioscler Thromb Vasc Biol.

[53] Beales IL, Ogunwobi OO (2010). Microsomal prostaglandin E synthase-1 inhibition blocks proliferation and enhances apoptosis in oesophageal adenocarcinoma cells without affecting endothelial prostacyclin production. Int J Cancer.

[54] Beales IL, Vardi I, Dearman L (2012). Regular statin and aspirin use in patients with Barrett’s oesophagus is associated with a reduced incidence of oesophageal adenocarcinoma. Eur J Gastroenterol Hepatol.

[55] Mokrowiecka A, Sokolowska M, Luczak E, Dudojc M, Wieczfinska J, Kacprzak D, Wierzchniewska-Lawska A, Pawliczak R, Malecka-Panas E (2013). Adiponectin and leptin receptors expression in Barrett’s esophagus and normal squamous epithelium in relation to central obesity status. J Physiol Pharmacol.

[56] Howard JM, Beddy P, Ennis D, Keogan M, Pidgeon GP, Reynolds JV (2010). Associations between leptin and adiponectin receptor upregulation, visceral obesity and tumour stage in oesophageal and junctional adenocarcinoma. Br J Surg.

[57] Miyashita T, Shah FA, Miwa K, Sasaki S, Nishijima K, Oyama K, Ninomiya I, Fushida S, Fujimura T, Hattori T, Harmon JW, Ohta T (2013). Impact of inflammation-metaplasia-adenocarcinoma sequence and prevention in surgical rat models. Digestion.

